# The Effects on Toxicity of Circadian Patterning of Continuous Hepatic Artery Infusion

**DOI:** 10.1155/1992/28702

**Published:** 1992

**Authors:** M. Margaret Kemeny, Galo Alava, Jorge M. Oliver, Fred B. Smith

**Affiliations:** 1 Department of Surgery St. Vincent’s Hospital and Medical Center 153 West 11th Street New York NY 10011 USA; 2 Department of Pathology St. Vincent’s Hospital and Medical Center 153 West 11th Street New York NY 10011 USA

## Abstract

Long term continuous hepatic artery infusion of FUDR was carried out in 34 rats. In the animals who received a constant infusion schedule of 15mg/kg/day all died of toxicity with a mean survival of 9.3 days. If the pattern of the continuous infusion was changed so that over 60% of the infusion was given during the hours of 3pm to 9pm then all of the animals survived the 14 day infusion. If the maximum dose of infusion was changed so that 60% of the infusion was given at night from 3am to 9am the infusion became more toxic and all the animals died in a mean of 5.5 days. Pathologic sectioning of all the livers reflected the above outcomes with the greatest amount of hepatic necrosis in the animals on the night cycles. This study underscores the recent advances in chronobiology demonstrating that for continuous hepatic arterial infusions the timing of delivery is crucial in determining the toxicity.


*HPB Surgery*, 1992, Vol. 5, pp. 185–194
Reprints available directly from the publisher
Photocopying permitted by license only

© 1992 Harwood Academic Publishers GmbH
Printed in the United Kingdom

### THE EFFECTS ON TOXICITY OF CIRCADIAN PATTERNING OF CONTINUOUS HEPATIC ARTERY INFUSION

M. MARGARET KEMENY*, GALO ALAVA*, JORGE M. OLIVER** and FRED B. SMITH**

*Department of Surgery* and Pathology**, St. Vincent's Hospital and Medical Center, 153 West 11th Street, New York, NY 10011, USA*

*(Received 16 May 1991)*

Long term continuous hepatic artery infusion of FUDR was carried out in 34 rats. In the animals who received a constant infusion schedule of 15mg/kg/day all died of toxicity with a mean survival of 9.3 days. If the pattern of the continuous infusion was changed so that over 60% of the infusion was given during the hours of 3pm to 9pm then all of the animals survived the 14 day infusion. If the maximum dose of infusion was changed so that 60% of the infusion was given at night from 3am to 9am the infusion became more toxic and all the animals died in a mean of 5.5 days. Pathologic sectioning of all the livers reflected the above outcomes with the greatest amount of hepatic necrosis in the animals on the night cycles. This study underscores the recent advances in chronobiology demonstrating that for continuous hepatic arterial infusions the timing of delivery is crucial in determining the toxicity.

KEY WORDS: Hepatic Artery infusion; circadian

### INTRODUCTION

Continuous hepatic artery infusion of FUDR has been studied in patients with colorectal metastases to the liver for the past 10 years. It is still the therapy that has the best response rates in these clinical situations, but many clinicians fear its use because of the severe and often fatal biliary toxicities associated with this treatment. Specifically biliary sclerosis, sometimes referred to as sclerosing cholangitis, can result after continuous hepatic artery infusion(CHAI) of floxuridine(FUDR)[1]. Although the complication is dose related, some patients develop biliary sclerosis even at the lowest dose of 0.1 mg/kg/d. Clearly a method to reduce this toxicity would enhance the usefulness of CHAI of FUDR. There has been a suggestion that using a circadian rhythm for the CHAI can lower the toxicity. Previous experiments were done in rats using an intravenous approach which did show a decrease of the toxicity of infusion for rats given a circadian pattern with the greatest amount given during the afternoon hours[2]. The present study utilizes a rat model which is identical to the human CHAI model. The rats all have hepatic artery catheters through which continuous infusions can be performed for 14 days, which is the same amount of time as the human perfusions. Using this model we studied the

Address correspondence to: M.Margaret Kemeny, M.D., F.A.C.S., Chief, Surgical Oncology, St. Vincent's Hospital, 153 West 11th Street, New York, NY 10011, USA

effects that changing flow rates of the FUDR had on the toxicity to the liver parenchyma.

### MATERIAL AND METHOD

Sixteen Sprague Dawley and 18 Fisher rats were kept in the St. Vincent's Medical Center animal facility, on rat chow, in an 8am to 8pm lights on and 8pm to 8am lights off routine. Each rat underwent a laparotomy for placement of a #10 Polyethylene tube(Clay Adams) for hepatic artery infusion. The average rat was 250 gm at the time of the operation and eight weeks old. The catheter was placed into the gastroduodenal artery and the artery was ligated distally. The catheter was then passed through the abdominal fascia, tunnelled under the skin to an opening between the scapulae. At that spot a metal button was placed subcutaneously with a coiled metal catheter attached (Instatech). The PE catheter was passed through the button into the coiled metal tube and was attached to a swivel placed above the rat's cage. This swivel was connected to the infusion pump. This arrangement allowed the rat free mobility in its cage, as well as protecting the button apparatus. For additional protection a small jacket was placed on the rat, preventing it from chewing on the catheter exit site.

The infusion pumps used were the portable Intelliject pumps with a computer programmable chip for a varied infusion cycle. The accuracy of flow rates were verified before use.

Eleven rats were placed on a constant infusion cycle of FUDR (Roche) at 15 mg/kg/day. The infusion was started 24 hours after the operation for placement of the catheter. Eleven rats were placed on a "day" cycle of infusion. In these animals a total dose of 15 mg/kg/day of FUDR was given in a volume of 4 cc/day. The infusion schedule was as follows: From 9am to 3pm 0.6cc was infused(0.1cc/hr), from 3pm to 9pm 2.7cc(0.45cc/hr) was infused, from 9pm to 3am 0.6cc(0.1cc/hr) was infused and from 3am to 9am 0.08cc(.013cc/hr) was infused (see Table 1). Twelve rats received 15 mg/kg/day of FUDR on a "night" cycle again in a volume of 4cc/d. These animals received 0.6cc from 9am to 3pm, 0.08cc from 3pm to 9pm, 2.7cc from 9pm to 3am and 0.6cc from 3am to 9am (see Table 2). All the infusions were given for 14 consecutive days or until the animal died if less than 14 days. The animals that survived the 14 day FUDR infusion were then infused with heparin and saline for 14 more days at which time they were sacrificed.

The livers of all the animals were excised and fixed in 10% neutral buffered formalin prior to pathological examination. All livers were sectioned at 3 mm intervals with no less than 2 blocks submitted for paraffin sections, which included any grossly visible lesion as well as representative hepatic parenchyma. A minimum of 4 $cm^2$ crosssectional area was examined histologically in each case. Hematoxylin and Eosin (H&E) stained sections were examined by two independent pathologists utilizing conventional light microscopy.

### RESULTS

All of the 11 animals on the constant infusion of FUDR died in 6 to 12 days with a mean survival of 9.3 days ± 1.7(Table 3). Seven of these animals were Fisher rats

**Table 1**

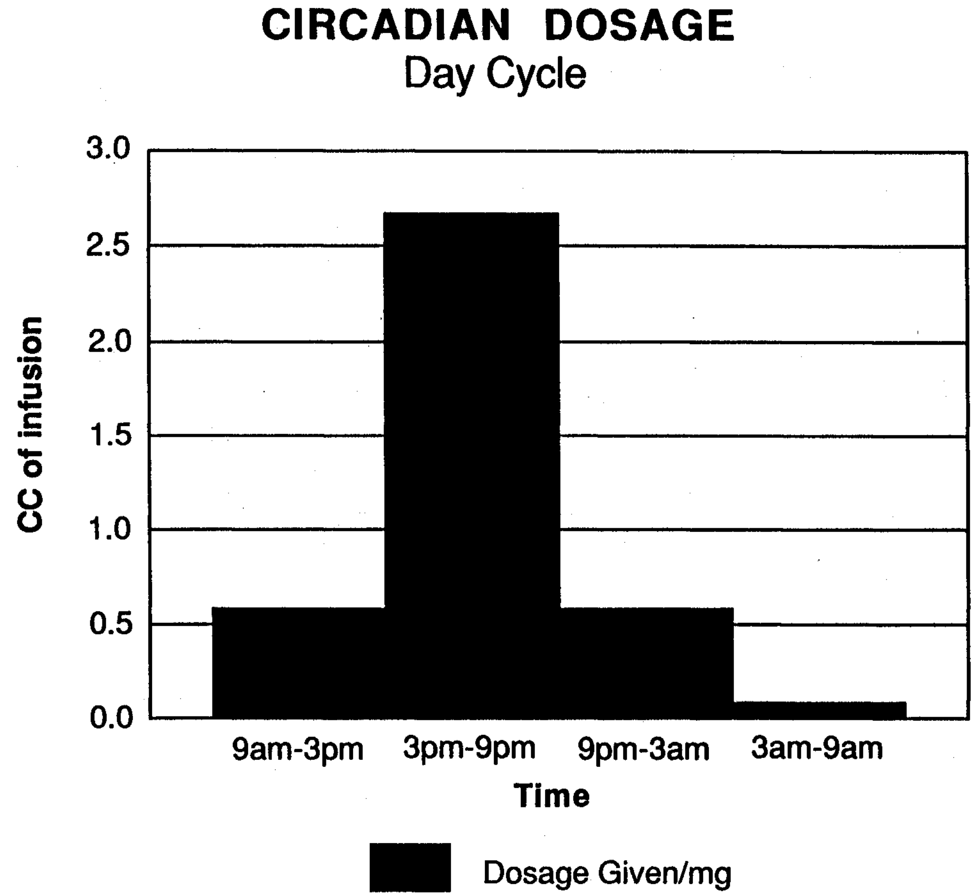

with a mean survival of 9.3 ± 1.8 days. The other 4 were Sprague Dawley rats with a mean survival of 9.25 ± 1.7 days. There was no significant difference in survival of these two strains. The rats began to look ill several days before their deaths. Most had mouth sores with ulcerations, eye infections and some bleeding per rectum.

All of the 11 animals who received the "day" cycle infusions survived the 14 days of infusion. One of the animals died one day later (day 15) of hemorrhagic colitis, while the 10 other animals were all sacrificed 15 days after the end of the 14 day infusion. None of these rats seemed ill, with no mouth ulcerations or eye problems.

In the "night" cycle infusion group all 12 animals died between 4 to 7 days with a mean survival of 5.5 ± 1.2 days. Again these rats seemed to have the same physical problems as the constant flow group with mouth ulcerations, eye infections and rectal bleeding. Half of this group were Fisher rats with a mean survival of 6.2 ± 1.2 days. The other half were Sprague Dawley rats with a mean survival of 4.8 ± 1. There was no statistically significant difference between the survival in these two strains.

**Table 2**

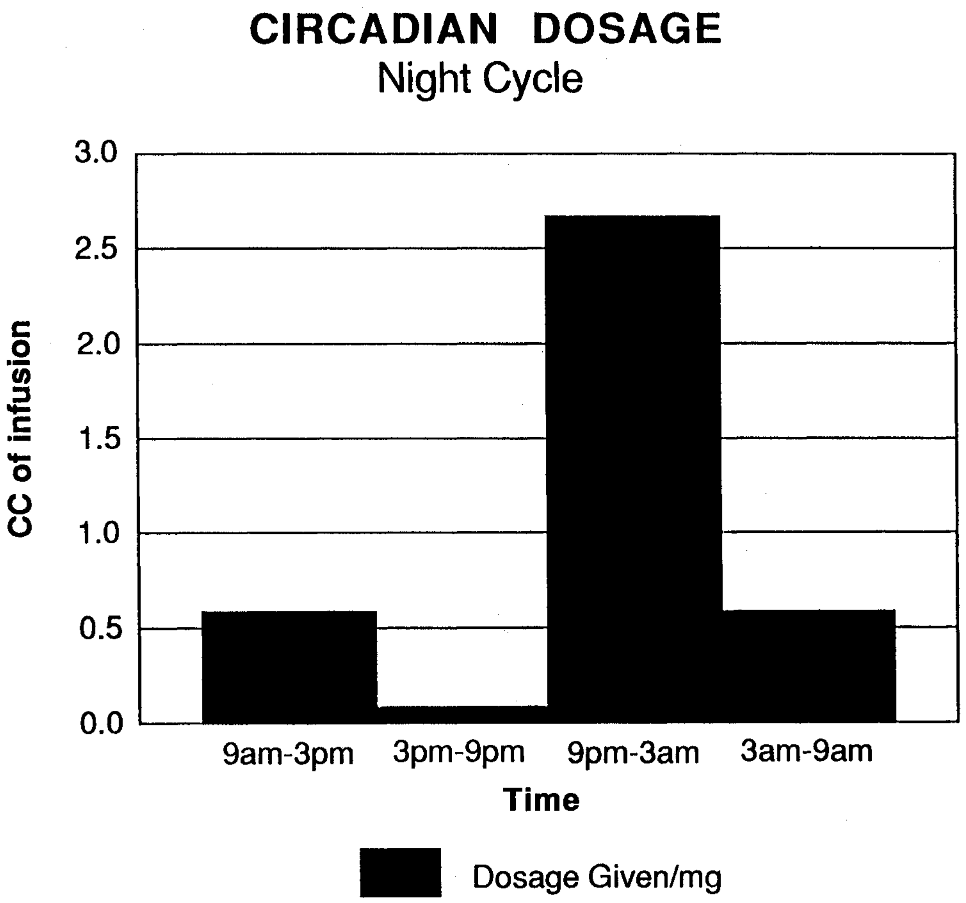

The group with the day cycle had the best response since none of the rats died of toxicity during the infusion. There was a statistically significant difference in the survival of the animals on the night cycle versus the constant infusion (Paired $t$-test $p < .0001$). The group treated with the night cycle had a poorer survival.

### PATHOLOGY

The livers from rats who had received a constant continuous infusion of FUDR were noted to contain rare portal chronic inflammatory cells and minimal centrilobular congestion as well as small degenerative foci within lobules. The general architecture appeared preserved with foci of single hepatocyte necrosis, swelling and vacuolization. Multifocal microvacuolar fatty changes were noted, predominantly in the centrilobular areas (Figure 1). For rats who had received a circadian timed day infusion of FUDR their livers had rare foci of hepatocyte swelling

**Table 3**

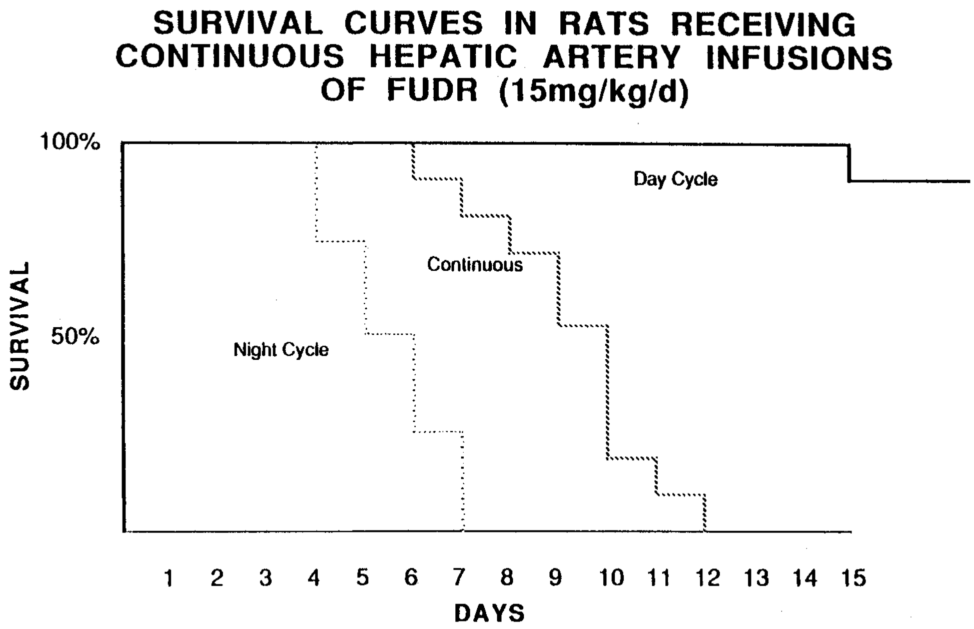

without evidence of vacuolar degeneration or single cell necrosis. No inflammatory infiltrates were noted (Figure 2). The livers in the rats who received a timed infusion of FUDR with the majority of the drug given at night had multifocal broad areas of necrosis some of which were extensive without identifiable zonal predilection. There was also extensive vacuolar degeneration with macrovacuolar fatty change predominantly in centrilobular areas. There was marked centrilobular chronic passive congestion. No significant lobular or portal inflammatory response was noted. A mild, diffuse, Kupffer cell hyperplasia was identified in those cases with extensive necrosis (Figure 3).

All livers lacked evidence of granulomas, eosinophilic inclusions, bile duct proliferation, sclerosis, fibrosis, bile plugs, cholestasis, vacuolar abnormalities or hepatocyte dysplasia.

### DISCUSSION

The concept that there are chemical changes in the body's ability to metabolize drugs that are dependent on a 24 hour or circadian rhythm is not new. Even scientists in the 15th century realized that there were physiologic changes that paralleled daily cycles. However this knowledge of chronobiology had not been utilized in the treatment of cancer with toxic chemotherapy until very recently. Now there are numerous clinical and animal studies showing that circadian timing of chemotherapy administration can decrease drug toxicity[3–8]. A study from France in 1987 showed that patients receiving a continuous infusion of 5-fluorouracil (5FU)

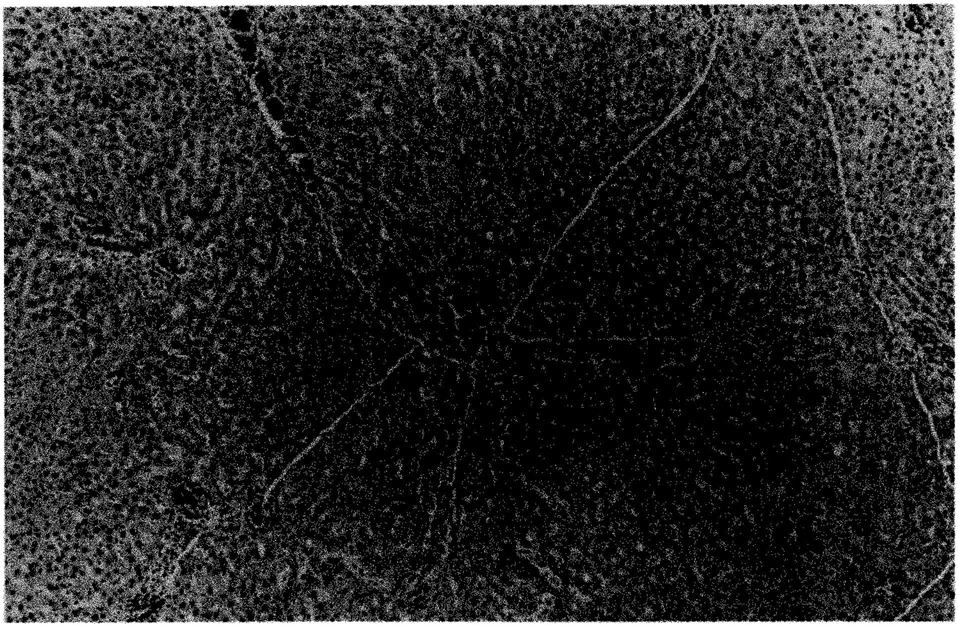

**Figure 1** This section from a liver of an animal receiving a constant continuous infusion of FUDR (Magnification 40×, H & E stain) shows a centrilobular area with hepatocyte swelling, vascular congestion, and foci of necrosis.

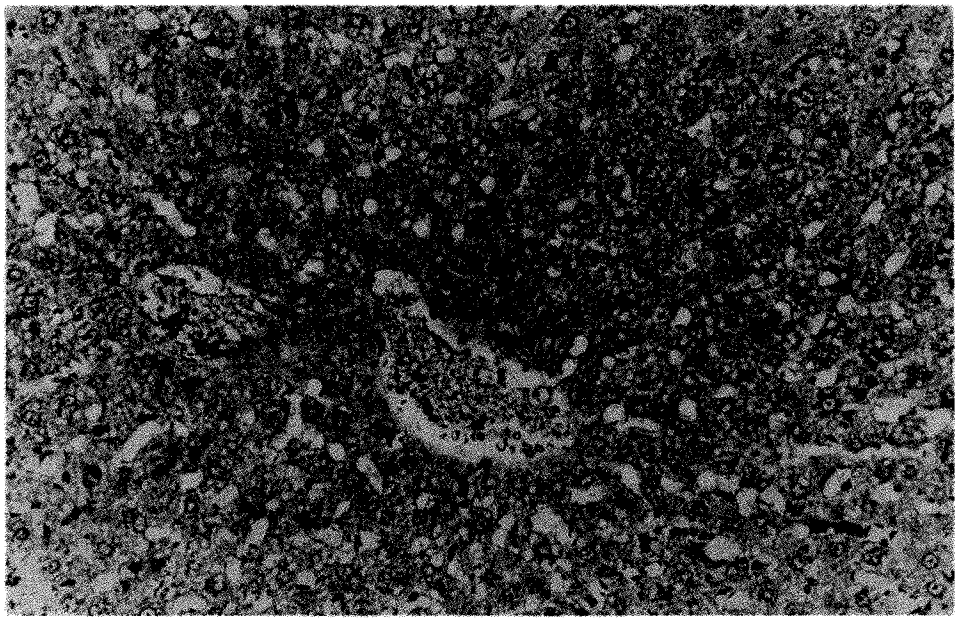

**Figure 2** This section from a liver of an animal receiving a circadian timed day infusion of FUDR (Magnification 470×, H & E stain) shows mild fatty and degenerative changes and vascular congestion.

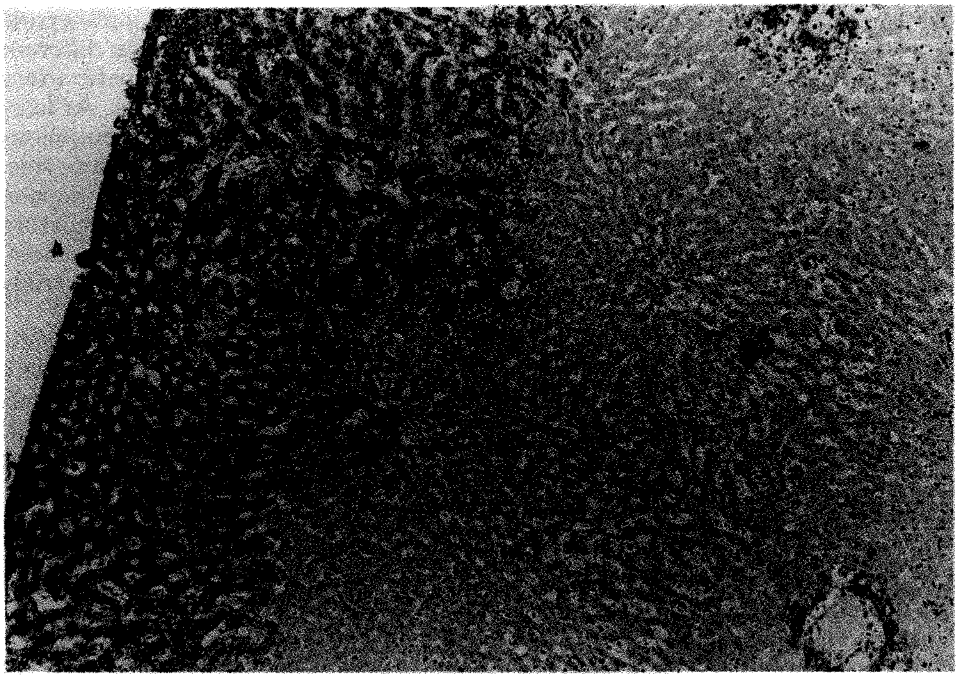

**Figure 3** This section from a liver of an animal receiving a circadian timed night infusion of FUDR (Magnification 40×, H & E stain) shows a peripheral section of liver with a broad zone of necrosis and fatty changes in non necrotic hepatocytes.

at a constant rate had varying plasma concentrations of the drug depending on the time of day. The lowest concentrations were seen during the daylight hours and the highest were at 12 midnight. There was a definite circadian pattern to the concentrations suggesting more absorption and metabolism during the day. Since the liver was the main organ of clearance of 5-FU this finding suggested a circadian pattern to hepatic metabolism[9].

When these concepts were utilized in patterning chemotherapy infusions new information on toxicity was obtained. A study of a continuous intravenous infusion of FUDR given to rats with a maximal daytime dose and minimal night dose was found to be less toxic than on a constant continuous infusion[2].

Toxicity is of key importance in the clinical setting when continuous hepatic artery infusion of FUDR is used for hepatic metastases. Implantable pumps have been used for over 10 years to get response rates of over 50% in patients with hepatic metastases from colorectal cancer[10,11]. No other modality at this time produces comparable responses in hepatic metastases. The main reason that hepatic infusion has not been universally adopted as the treatment of choice for colorectal metastases to the liver is because of the incidence of biliary sclerosis associated with therapy. It is now felt that approximately 20% of patients getting CHAI will develop biliary sclerosis[1,10,11]. Consequently, decreasing this incidence of toxicity would greatly enhance the value of CHAI of FUDR. Previous experiments in rats have shown that intravenous FUDR given by a continuous constant flow was more toxic than the same drug given in a circadian daytime pattern[2,8,12]. Diarrhea was the toxicity being studied. This work was then extrapolated to the intrahepatic artery infusions of FUDR. However the intraarterial situation is quite different since biliary sclerosis, not diarrhea, is the toxicity. Also, infusing the hepatic artery is anatomically unique because of the dual blood supply of hepatocytes. At least 70% of hepatic afferent flow comes from the portal vein; hence, a continuous hepatic artery infusion may not have a strong impact on normal hepatocytes. However, hepatic neoplasms derive 90% of their blood supply from the hepatic artery, not the portal vein. Shunting may occur into the normal hepatic vasculature exposing more hepatocytes to the continuous infusion. Because of this uniqueness, an animal model of continuous hepatic infusion of FUDR was needed to study toxicity.

In this study a rat model was used where the animals were infused via an hepatic artery catheter for the same 14 day cycle that is used for patients. When rats were infused with CHAI of FUDR at doses 50 times the level of human doses the rats suffer a toxic death which is marked by hepatic necrosis, diarrhea and mucositis. If the same dose is given by use of circadian patterning with over 50% of the dose delivered betwee 3pm and 9pm in the afternoon none of the rats died. With this minimal change in flow patterning there is a dramatic decrease in toxicity. When the circadian patterning was switched to a cycle where most of the FUDR was given at night (night cycle) not only did all the animals die but they died in a statistically significantly shorter interval than the animals receiving a constant rate infusion.

This study underscores the importance of circadian timing especially in continuous infusional therapy. A uniformly fatal dose of FUDR can be tolerated if the drug is given on what we term a day cycle. Survival and liver pathology are greatly improved with this type of cycling. In addition, it appears that night cycling is more toxic than a flat rate; further evidence supporting the concept of timing.

The exact mechanism of this response is unclear but it suggests that there may be circulating factors which are dependent on diurnal variation. Steroids would certainly be a consideration since they are known to increase hepatic catobolism of toxic substances.

The next step is to look at this patterning of FUDR in a tumor bearing host to evaluate if the decreased toxicity is a trade off for decreased tumor response. Experiments are underway to test this hypothesis.

*Acknowledgements*

The financial support of Medtronics and Pfizer-Infusaid is gratefully acknowledged.

*References*

1. Kemeny, M.M., Battifora, H., Blayney, D.W., Cecchi, G., Goldberg, D.A., Leong, L.A., Margolin, K.A. and Terz, J.J. (1985) Sclerosing cholangitis after continuous hepatic artery infusion of FUDR. *Ann.of Surg.* **202**, 176–181
2. Roemeling, R.V., Mormont, M.C., Walker, K. *et al.* (1987) Cancer control depends upon the circadian shape of continuous FUDR infusion. *Proc.Am.Assoc.Cancer Res*, **28**, 326 (abstract 1293)
3. Hrushesky, W.J.M. (1985) Circadian Timing of Cancer Chemotherapy. *Science*, **228**, 73–75
4. Damascelli, B., Marchiano, A., Spreafico, C. *et al* (1990) Circadian continuous chemotherapy of renal cell carcinoma with an implantable, programmable infusion pump. *Cancer*, **66**, 237–241
5. Sothern, R.B., Levi, F., Halberg, H.F. and Hrushesky, W.J.M. (1989) Control of a murine plasmacytoma with doxorubicin-cisplatin: Dependence on circadian stage of treatment. *J.Nat.Cancer Inst.* **81**, 135–145
6. Hrushesky, W.J.M., von Roemling, R., Lanning, R.M. and Rabatin, J.T. (1990) Circadian-shaped infusions of floxuridine for progressive metastatic renal cell carcinoma. *J.of Clin.Oncol.* **8**, 1504–1513
7. Roemeling, R., Hrushesky, W.J.M., Remick, S., Horton, J. *et al.* (1990) Continuous intravenous, variable rate FUDR infusion: A randomized pilot study comparing two circadian schedules for toxicity and maximally tolerated dose intensity. *Proc. Am. Soc Clin ONCOL,* **9**, 85 (abstract 329)
8. von Roemeling, R., Hrushesky, W.J.M. (1989) Circadian patterning of continuous floxuridine infusion reduces toxicity and allows higher dose intensity in patients with widespread cancer. *J.Clin.Oncol.* **7**, 1710–1719
9. Petit, E., Milano, G., Levi, F., Thyss, A., Bailleul, F. and Schneider M. (1988) Circadian rhythm-varying plasma concentration of 5-Fluorouracil during a five day continuous venous infusion at a constant rate in cancer patients. *Cancer Research,* **48**, 1676–1679
10. Kemeny, M.M., Goldberg, D., Beatty, J.D., Blayney, D. *et al.* (1986) Results of a prospective randomized trial of continuous regional chemotherapy and hepatic resection as treatment of hepatic metastases from colorectal primaries. *Cancer,* **57**, 492–498
11. Kemeny, N., Daly, J., Reichman, B., Geller, N., Botet, J. and Oderman, P. (1987) Intrahepatic or systemic infusion of fluorodeoxyuridine in patients with liver metastases from colorectal carcinoma. *Ann.of Intern.Med.,* **107**, 459–465
12. Wesen, C., Olson, G., Roemeling, R., Grage, T. and Hrushesky, W.J.M. (1989) Circadian modified intra-arterial treatment of colorectal carcinoma metastatic to the liver allows higher dose intensity to be safely given. *ASCO* Vol 8, March 1989

*(Accepted by S.Bengmark 24 July 1991)*

### INVITED COMMENTARY

This study corroborates previous findings that circadian timing of cytostatic drug administration may affect toxicity. This is an interesting observation that may be utilized in the clinical setting.

In the present study toxicity may have been lower at daytime than at night because of (1) increased catabolism of the drug, (2) altered effect(s) of the drug at the cellular level or (3) a relatively high ability to repair cellular damage. Petit *et al.* demonstrated that plasma concentrations of 5-fluorouracil (5-FU) were higher at night than at daytime in patients receiving a continuous infusion for 5 days[1], indicating that there is a diurnal variation of the metabolism of 5-FU.

Increased antineoplastic activity and reduced toxicity are crucial elements in cytostatic drug therapy. A possible way to increase selectivity is to administer the drug at a time of the day when tolerance is relatively good, an approach made practical by the development of implantable and programmable infusion systems. However, circadian patterning of drug infusion is going to be advantageous only if there is a difference between neoplastic and normal cells with respect to one or more of the possible mechanisms for altered toxicity. It appears reasonable to assume that malignant cells are less susceptible to circadian changes than normal cells, and this has been proven in animal models[2]. In addition, there may be a circadian variation in the effect of cytostatic drugs on malignant cells. Thus, it has been shown that there is a marked, dose-independent circadian difference in the ability of doxorubicin-cisplatin or FUDR to produce tumour response and cure in rats with transplanted plasmacytoma or adenocarcinoma[2,3].

There is clearly a need for continued studies on the benefits of circadian timing in cancer chemotherapy. Further studies in animals and, in particular, in patients are awaited with interest.

### *References*

1. Petit, E., Milano, G., Levi, F., Thyss, A., Bailleul, F. and Schneider, M. (1988) Circadian rhythm-varying plasma concentration of 5-fluorouracil during a five day continuous venous infusion at a constant rate in cancer patients. *Cancer Res.*, **48**, 1676–1679
2. Sothern, R.B., Levi, F., Haus, E., Halberg, F. and Hrushesky, W.J.M. (1989) Control of a murine plasmacytoma with doxorubicin-cisplatin: dependence on circadian stage of treatment. *J.Natl.Cancer Inst.*, **81**, 135–145
3. Roemeling, R., Hrushesky, W.J.M. (1989) Circadian patterning of continuous floxuridine infusion reduces toxicity and allows higher dose intensity in patients with widespread cancer. *J.Clin.Oncol.*, **7**, 1710–1719

Karl-G Tranberg
Department of Surgery
Lund University
Lund
Sweden

